# Identification of Non-Coding RNAs in the *Candida parapsilosis* Species Group

**DOI:** 10.1371/journal.pone.0163235

**Published:** 2016-09-22

**Authors:** Paul D. Donovan, Markus S. Schröder, Desmond G. Higgins, Geraldine Butler

**Affiliations:** 1 School of Biomedical and Biomolecular Science and UCD Conway Institute of Biomolecular and Biomedical Research, Conway Institute, University College Dublin, Belfield, Dublin 4, Ireland; 2 School of Medicine and UCD Conway Institute of Biomolecular and Biomedical Research, University College Dublin, Belfield, Dublin 4, Ireland; Vanderbilt University, UNITED STATES

## Abstract

The *Candida* CTG clade is a monophyletic group of fungal species that translates CTG as serine, and includes the pathogens *Candida albicans* and *Candida parapsilosis*. Research has typically focused on identifying protein-coding genes in these species. Here, we use bioinformatic and experimental approaches to annotate known classes of non-coding RNAs in three CTG-clade species, *Candida parapsilosis*, *Candida orthopsilosis* and *Lodderomyces elongisporus*. We also update the annotation of ncRNAs in the *C*. *albicans* genome. The majority of ncRNAs identified were snoRNAs. Approximately 50% of snoRNAs (including most of the C/D box class) are encoded in introns. Most are within mono- and polycistronic transcripts with no protein coding potential. Five polycistronic clusters of snoRNAs are highly conserved in fungi. In polycistronic regions, splicing occurs via the classical pathway, as well as by nested and recursive splicing. We identified spliceosomal small nuclear RNAs, the telomerase RNA component, signal recognition particle, RNase P RNA component and the related RNase MRP RNA component in all three genomes. Stem loop IV of the U2 spliceosomal RNA and the associated binding proteins were lost from the ancestor of *C*. *parapsilosis* and *C*. *orthopsilosis*, following the divergence from *L*. *elongisporus*. The RNA component of the MRP is longer in *C*. *parapsilosis*, *C*. *orthopsilosis* and *L*. *elongisporus* than in *S*. *cerevisiae*, but is substantially shorter than in *C*. *albicans*.

## Introduction

Approximately 9.5% of nosocomial infections are caused by pathogenic fungi, such as *Aspergillus fumigatus*, *Cryptococcus neoformans* and members of the *Candida* CTG clade [1, 2]. CTG-clade species, which translate the CTG codon as serine rather than leucine, include major human fungal pathogens such as *Candida albicans*, *Candida tropicalis* and *Candida parapsilosis*, and the xylose-degrading species *Spathaspora passalidarum*, *Scheffersomyces stipitis* and *Candida tenuis* [3–6]. *C*. *albicans* is responsible for the majority of *Candida* infections. However, its incidence has decreased in recent years accompanied by increased prevalence of other *Candida* species, including *C*. *tropicalis* and *C*. *parapsilosis* [7–9].

The genomes of several pathogenic and non-pathogenic CTG species have been sequenced, and at least partly annotated [10–14]. Most annotation efforts have concentrated on protein coding genes. For example, the Candida Gene Order Browser (CGOB; [5, 15]) used manual curation together with sequence similarity and synteny information to improve protein prediction in 13 CTG clade species. This approach identified >1,500 new genes, and highlighted errors in automated annotation such as the surprisingly high number of introns initially predicted in the genome of *S*. *stipitis* [5]. However, apart from identification of tRNAs using tRNAscan-SE [16], there has been very little emphasis placed on identifying ncRNAs in CTG-clade species.

After tRNAs, the most common ncRNAs in *Candida* genomes are small nucleolar RNAs (snoRNAs). snoRNAs guide the nucleotide modifications of other ncRNAs, including ribosomal RNAs and tRNAs. There are two main types of snoRNAs; C/D box and H/ACA box snoRNAs. C/D box snoRNAs contain conserved motifs (C and D boxes) and guide the methylation of RNA nucleotides. These snoRNAs are around 100 nucleotides in length. H/ACA box snoRNAs guide the pseudouridylation of RNA nucleotides. These snoRNAs also contain conserved motifs, (H and ACA boxes), but are better distinguished by their conserved secondary structures. H/ACA snoRNAs are generally longer (over 150 nucleotides) than C/D box snoRNAs. Each snoRNA must associate with a number of proteins forming the small nucleolar ribonucleoprotein complex before they can modify RNA nucleotides.

The *C*. *albicans* genome is by far the best characterized of the *Candida* species [10, 11, 17]. Two studies in particular used a combination of bioinformatics and experimental analyses to identify the non-coding landscape in this species [18, 19]. Sellam et al [19] identified 27 C/D box and 35 H/ACA box snoRNAs, the long spliceosomal form of the U5 small nuclear RNA, the telomerase RNA component and the RNase MRP RNA component. Mitrovich et al [18] identified 40 C/D box snoRNAs, and showed that the majority are found in intronic regions. This is very different to snoRNA organization in *Saccharomyces cerevisiae*, where only six C/D box snoRNAs are intronic [20].

Here, we use both bioinformatics analysis and experimental evidence (RNA-seq) to analyze the ncRNA content in genomes from *C*. *parapsilosis*, and its close relatives *Candida orthopsilosis and Lodderomyces elongisporus* [21]. *C*. *orthopsilosis* is also a pathogen, but is more rarely isolated from patient samples [22]. *L*. *elongisporus* is not generally considered a human pathogen [23], although it has been identified in a small number of patient samples [24]. We identify snoRNAs, small nuclear RNAs (snRNAs) that primarily function in the splicing of pre-mRNAs and the RNA components of a number of ribonucleoprotein complexes. We also updated the ncRNA predictions for the *C*. *albicans* genome. We find that like *C*. *albicans*, a large proportion of C/D box snoRNAs in *C*. *parapsilosis*, *C*. *orthopsilosis* and *L*. *elongisporus* are intronic, and a greater proportion of H/ACA box snoRNAs are exonic. In addition, our analyses greatly improves the available annotations of *Candida* genomes, which will facilitate the future identification of novel long ncRNAs, such as those involved in regulatory processes [25].

## Results and Discussion

### Identification of ncRNAs

We used several approaches to identify ncRNAs in *C*. *parapsilosis*, *C*. *orthopsilosis* and *L*. *elongisporus*. We first extracted 211 ncRNA features (not including tRNAs and rRNAs) from the *S*. *cerevisiae* and *C*. *albicans* genomes [26, 27] and compared them to the *C*. *parapsilosis* genome. 32 ncRNAs were identified in *C*. *parapsilosis*, comprising mostly of snoRNAs (S1 Table). The relative lack of success suggests that the primary sequence of ncRNAs is poorly conserved between *S*. *cerevisiae*, *C*. *albicans* and *C*. *parapsilosis*. However, ncRNAs are likely to retain some sequence conservation in more closely related species, that is, ncRNAs that lie in the so-called “Goldilocks’ zone” [28]. We also predicted that the location of ncRNAs would be syntenically conserved between closely related species. We therefore extracted syntenic intergenic regions from CGOB ([5, 15]) and identified conserved regions using BLAST. ncRNAs in these regions were identified by comparison to known sequences in *C*. *albicans* and *S*. *cerevisiae*, and by similarity to NCBI BLAST databases. 64 ncRNAs were identified in *C*. *parapsilosis* using this method.

We next modified our approach by using the pattern-scanning programs Snoscan and snoGPS to specifically identify C/D box and H/ACA box snoRNAs, respectively [29, 30]. Snoscan predicted 24 C/D box (methylation-guide) snoRNAs and snoGPS predicted 9 H/ACA box (pseudouridylation-guide) snoRNAs in *C*. *parapsilosis*. Thirdly, we used Infernal with the RFAM covariance models, a method that has recently emerged as a more sensitive and accurate system for ncRNA identification [31, 32]. After removal of tRNAs, rRNAs and unlikely candidates, approximately 47 ncRNAs were predicted in the *C*. *parapsilosis* genome. The same methods were then applied to *C*. *orthopsilosis* and *L*. *elongisporus*, adding *C*. *parapsilosis* ncRNAs to the BLAST comparisons.

We supported the bioinformatics analysis with experimental evidence. We used RNA-seq to characterize the transcriptome of *C*. *parapsilosis*, *C*. *orthopsilosis* and *L*. *elongisporus* growing in rich media. ncRNA predictions that were not supported by transcriptional evidence were removed from further analyses.

**Fig 1:**
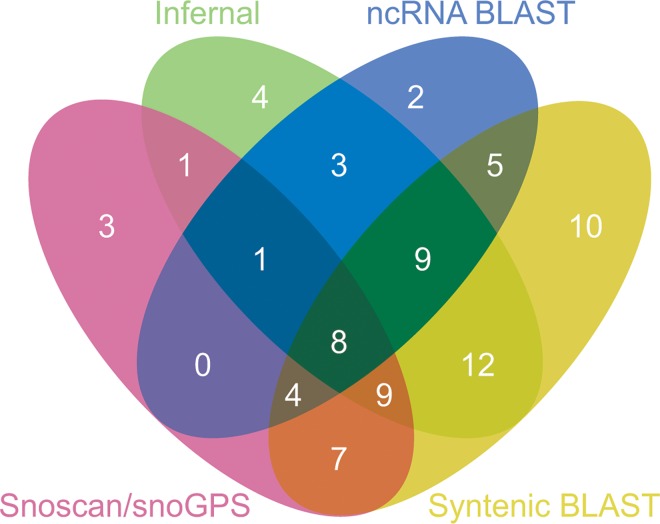
Identification of ncRNAs in *C*. *parapsilosis*. Only ncRNAs supported by RNA-seq data or by manual curation are shown.

A comparison of the various approaches is shown in Fig 1. The syntenic BLAST approach identified the most candidates. However, this approach, together with the BLAST analysis of known ncRNA features, identified only sequence fragments, particularly of snoRNAs. Snoscan and snoGPS identified full-length orthologs of *S*. *cerevisiae* snoRNAs. Infernal was the most efficient method for identifying full-length ncRNAs.

### Analysis of ncRNAs

Apart from tRNAs, 78 ncRNAs were identified in *C*. *parapsilosis* and *L*. *elongisporus*, and 77 in *C*. *orthopsilosis* (Table 1 and S1 Table), including the signal recognition particle RNA component (SCR1), part of a ribonucleoprotein complex responsible for the translocation of newly synthesized (or mid-synthesis) proteins from the ribosome to the endoplasmic reticulum. A typical eukaryotic SCR1 is 300 nucleotides in length [33]. The SCR1s in the *Candida* species range from 265 nucleotides in *C*. *albicans* to 300 nucleotides in *L*. *elongisporus*. All three of the species examined have a conserved large (S) domain, with a more variable small (Alu) domain (S1 Fig). The S domain, together with associated proteins, interacts with the SCR1 receptor in the membrane of the endoplasmic reticulum, whereas the small (Alu) domain associates with the ribosome [34]. Variations in SCR1 size and secondary structure have been described in other Ascomycota species. In *S*. *cerevisiae* for example, the SCR1 is 522 nucleotides and contains additional helices that stem from the Alu domain [33].

**Table 1:** Identification of ncRNAs in CTG-clade *Candida* species and *S*. *cerevisiae*.

Non-coding RNA	*C*. *parapsilosis*	*C*. *orthopsilosis*	*L*. *elongisporus*	*C*. *albicans*	*S*. *cerevisiae*
tRNAs	91	81	106	126	299
C/D box snoRNAs	48	47	48	47 (45*)	47
H/ACA box snoRNAs	21	21	21	21 (30*)	29
Small Nuclear RNAs	5	5	5	5**	5**
Signal Recognition Particle RNA	1	1	1	1	1
Telomerase RNA	1	1	1	1	1
RNase MRP	1	1	1	1	1
RNase P	1	1	1	1	1

rRNA data is taken from SGD [26], CGD [27], and CGOB [5].

*C. *albicans* snoRNA numbers were taken from CGD [27], Final predicted snoRNA numbers are presented without parentheses. See S1 Table for complete information.

**Both *S*. *cerevisiae* and *C*. *albicans* have two isoforms of snR5, long and short versions. It is likely that the other three species also contain these.

The RNA component of telomerase (TER1), which acts as a template for telomere formation, is known to evolve rapidly [35]. TER1 was identified in the three species analyzed by comparison with C. *albicans* TER1 (S1 Table). The primary sequence however is less conserved than for snoRNAs. The orthologs of RNase P and RNase MRP were also identified. RNaseP is a ribozyme that cleaves precursor tRNA molecules, whereas RNase MRP cleaves the internal transcribed spacer 1 between the 5.8S and 18S rRNAs. Although functionally distinct, RNase P and RNase MRP require the same core subunits to become catalytically active [36]. Piccenelli et al [37] found that the *C*. *albicans* RNase MRP was much longer than other fungal MRPs (2,226 nt compared to 339 nt in *S*. *cerevisiae*), associated with a large insertion. Domain 2 of RNase MRP is particularly variable in fungi, but the *Candida* clade is an extreme case [38]. The RNase MRP orthologs in *C*. *parapsilosis*, *C*. *orthopsilosis* and *L*. *elongisporus* are 991 nt, 971 nt and 907 nt respectively. The 5’ and 3’ regions of RNase MRP are relatively well conserved between these species. However, the middle of the RNase MRP molecule is more divergent (S1 File). The 5’ and 3’ ends of RNase MRP likely correspond to domain 1, and the mid-section to domain 2. Considering the level of conservation observed in protein-coding genes, the evolution of ncRNAs such as the SCR1 and RNase MRP is remarkably rapid.

Five small nuclear RNAs (U1, U2, U4, U5, U6), which primarily function in the splicing of introns from mRNA molecules, are highly conserved as previously described [39]. These ncRNAs form part of the spliceosome, a large ribonucleoprotein complex. Although the majority of U2 orthologs have the same secondary structure, in *C*. *parapsilosis* U2 has a deletion in stem loop IV [40]. U2A and U2B (Lea1 and Msl1 in *S*. *cerevisiae*, respectively) which bind to stem loop IV are also absent from *C*. *parapsilosis* [40]. We find that *C*. *orthopsilosis* but not *L*. *elongisporus*, share the stem loop IV deletion, and have lost the associated proteins U2A and U2B. The structures are also lost from *Candida metapsilosis* suggesting that the loss occurred in the ancestor of *C*. *parapsilosis*, *C*. *orthopsilosis* and *C*. *metapsilosis*, after the split with *L*. *elongisporus* [41, 42]. The structure and function of the spliceosome is therefore likely to be different in the *C*. *parapsilosis* species complex in comparison with other closely related *Saccharomycotina* species.

### Small nucleolar RNAs

The majority of ncRNAs identified are snoRNAs (Table 1). The numbers of C/D box snoRNAs are similar in *C*. *parapsilosis*, *C*. *orthopsilosis* and *L*. *elongisporus*, and in *C*. *albicans* and *S*. *cerevisiae* (Table 1). The number of predicted H/ACA box snoRNAs is somewhat lower in the first three species. The annotations in *S*. *cerevisiae* are taken from SGD [26]. In *C*. *albicans*, H/ACA box snoRNAs were predicted by Sellam et al [19] using snoGPS. We find that this method, which is based on *S*. *cerevisiae* sequences, tends to over-predict in *Candida* species. For example, the predicted snR189c in *C*. *albicans* overlaps with the 3’ splice site of the intron containing snR69. Orthologs of snR189c were not predicted in any of the other species analyzed. We also failed to identify orthologs of *S*. *cerevisiae* H/ACA box snoRNAs snR9, snR33, snR34, snR81, snR83, snR84, snR85, snR86, and C/D box snoRNAs snR39 and snR59 in *C*. *parapsilosis*, *C*. *orthopsilosis* or *L*. *elongisporus*. In contrast, the C/D box snoRNAs CD39, LSU-C2809 and LSU-G1431 are found in all *Candida* species examined but not in *S*. *cerevisiae* (CD39 was first identified in *Neurospora crassa* [43]; both LSU snoRNAs were first identified in *C*. *albicans* [39]). We have updated the ncRNA annotations in *C*. *albicans*, previously described by two groups [19, 39] (full detail in S1 Table). This includes the addition of 11 H/ACA box and two C/D box snoRNAs, and the removal of 20 H/ACA box snoRNAs.

**Fig 2:**
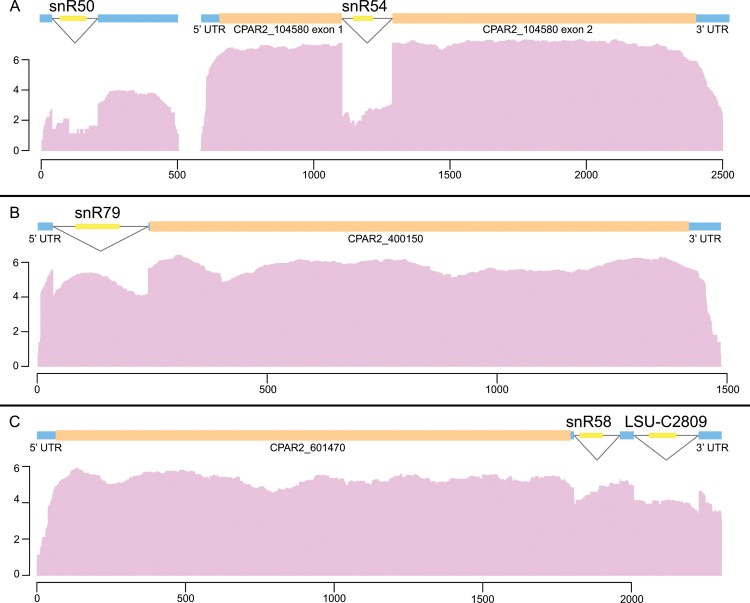
Location of snoRNA genes in *Candida* species. Examples shown are from *C*. *parapsilosis*. Open reading frames are indicated by orange bars and non-coding exons and untranslated regions of protein-coding genes by blue bars. snoRNAs are shown in yellow, and introns are shown with a black line. RNA-seq coverage is shown in pink. Nucleotides are numbered on the x-axis, and log transcription values on the y-axis. **(A)** C/D box snoRNA snR50 is located in an intron of a non protein-coding transcript. C/D box snoRNA snR54 is located in an intron within a protein-coding gene. **(B)** C/D box snoRNA snR79 is located in an intron in the 5’ UTR of a protein-coding gene. (C) Two snoRNAs in 3’ UTR of CPAR2_601470.

Approximately 50% of *Candida* snoRNAs are found in introns of any type; 37 of 68 in *C*. *albicans*, 34 of the 69 in *C*. *parapsilosis*, 33 of 68 in *C*. *orthopsilosis*, and 36 of 69 in *L*. *elongisporus* (S1 Table). A small number of snoRNAs are found in introns associated with protein-coding genes. In *C*. *albicans* and *L*. *elongisporus*, eight snoRNAs are found in introns within ORFs, of which seven are conserved in *C*. *parapsilosis* and *C*. *orthopsilosis* (S1 Table). The H/ACA box snR191 is located in an intron within the *NOG2* ortholog in *C*. *albicans*, *L*. *elongisporus* and *S*. *cerevisiae*. Although *C*. *parapsilosis* and *C*. *orthopsilosis* have introns in *NOG2*, they are too short to encode snR191. Instead, snR191 in encoded by a monocistronic transcript that does not contain an intron. A second H/ACA box snoRNA, snR44, is located inside an intron of the RPS22A ortholog in *C*. *albicans*, *L*. *elongisporus*, *C*. *parapsilosis* and *C*. *orthopsilosis* and is the only H/ACA box snoRNA located in an intron within a protein coding gene in the latter two species. CPAR2_601470 (encoding a Putative mitochondrial ATP-dependent RNA helicase) was previously shown to have two introns in the 3’ UTR [44]. We find that each intron contains a snoRNA (snR58 and LSU-C2809) (Fig 2). The same organization is found in *C*. *orthopsilosis*, *L*. *elongisporus* and *C*. *albicans*. One snoRNA (snR79) is located in a 5’ UTR in all four of these species (Fig 2B).

**Fig 3:**
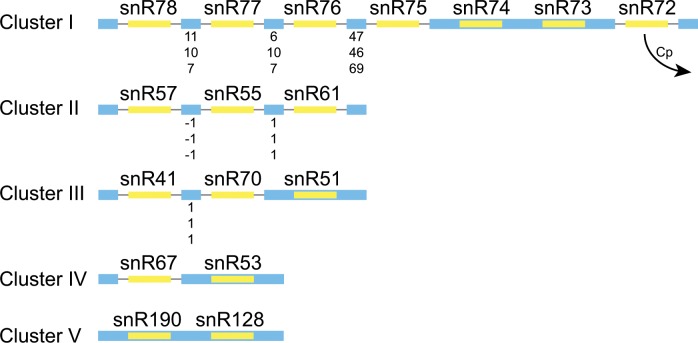
Conservation of polycistronic snoRNA clusters. Exons are shown in blue, and introns are shown with a black line. snoRNAs are shown in yellow. Exon lengths are shown in descending order for *C*. *parapsilosis*, *C*. *orthopsilosis* and *L*. *elongisporus*, respectively. Figure is not to scale. Some species have minor differences, including the loss of snR72 from cluster I in *C*. *parapsilosis*.

The majority of snoRNAs are found within mono- and polycistronic transcripts with no protein coding potential (e.g. snR50, Fig 2). Many of the polycistronic transcripts contain snoRNAs both in exonic sequences, and within spliced introns (e.g. cluster I, Fig 3). We identified five polycistronic regions shared by *L*. *elongisporus*, C. *parapsilosis*, *C*. *orthopsilosis* and *C*. *albicans* that encode 19 snoRNAs in each genome (Fig 3). Three of the polycistronic clusters were previously described in *C*. *parapsilosis* and *L*. *elongisporus* [45]. Clusters I, II and III are almost completely conserved in species in the Saccharomycotina and are strongly conserved in most of the Ascomycota and the Basidiomycota [43, 45–47]. Cluster V is also conserved in *N*. *crassa* [43, 47].

**Fig 4:**
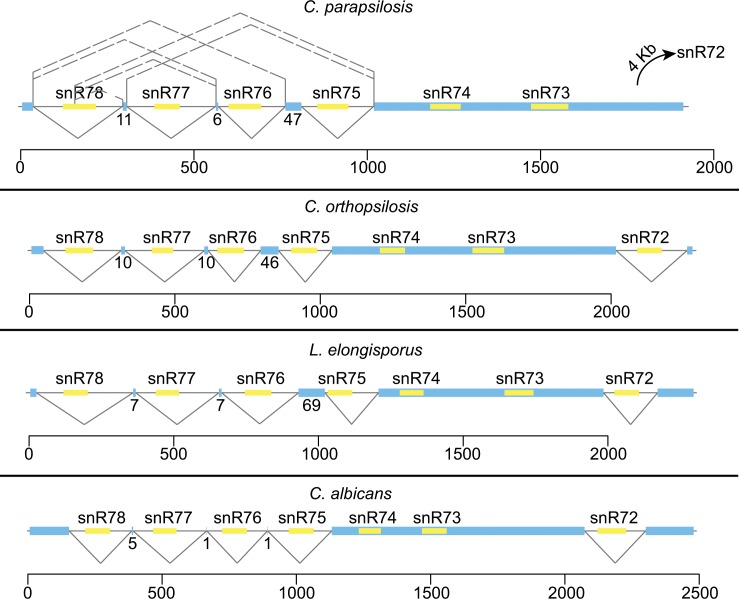
Alternative splicing of snoRNAs in Cluster I. Blue bars represent non-protein-coding exons with exon lengths indicated below. snoRNAs are shown in yellow. Introns are shown as black lines. Alternative splicing is depicted using broken lines. The major splice products in *C*. *parapsilosis* are shown below the line, with minor splice products shown above. Nucleotides are numbered on the x-axis, and log transcription values on the y-axis.

In *C*. *parapsilosis*, the intergenic region containing cluster I is smaller (2,367 bp) than the equivalent region in the other *Candida* species (e.g. 4,411 bp in *C*. *orthopsilosis*). This is because in *C*. *parapsilosis* snR72 is not found in this cluster, and instead is located 4 kb downstream within an intron in a new monocistronic transcript (Fig 4). snR72 is also absent from cluster I in some other *Saccharomycotina* species such as *Yarrowia lipolytica* and *Meyerozyma guilliermondii*, in the Pezizomycotina and in many Basidiomycetes [45]. Luo et al [45] suggests that these changes in location occur via an “excision-and-insertion” model, whereby the intron is excised entirely (or almost entirely) from the original location and is inserted at a staggered double-stranded break at a new locus. In *Y*. *lipolytica* and the *Pezizomycotina*, the snR72 intron recombined with the snR78 intron. In *C*. *parapsilosis*, the excised fragment was inserted some distance away from the cluster.

Mitrovich et al [39] have previously shown that processing of snoRNAs from polycistronic clusters in *C*. *albicans* involves alternative splicing. Splicing patterns in *C*. *parapsilosis* were investigated using RNA-seq, incorporating some data from Holland et al. [48]. The splice variants observed for cluster I are shown in Fig 4. The most common pattern observed is that all four introns are individually spliced. In minor spliced variants, several of the intervening exons are skipped.

An unusual splice event was observed within snR78. A consensus 5’ splice site is present in this position in *C*. *parapsilosis*, *C*. *orthopsilosis* and *L*. *elongisporus*, but not in *C*. *albicans* (GTATGT versus GTCTGT). It is unlikely that splicing at this position produces a viable snR78 molecule. All other 5’ splice sites match the consensus, and all 3’ splice sites contain either TAG or CAG sequences [18]. All but one intron has the standard TACTAAC branch site (GACTAAC in the snR75 intron).

Similar alternative splicing (except for within snR78) have been described in *N*. *crassa* [43]. It is likely that comparable splicing occurs in the orthologous regions in *C*. *orthopsilosis* and *L*. *elongisporus*. However, the read depth is lower from these species, and it was not possible to identify minor spliced products.

**Fig 5:**
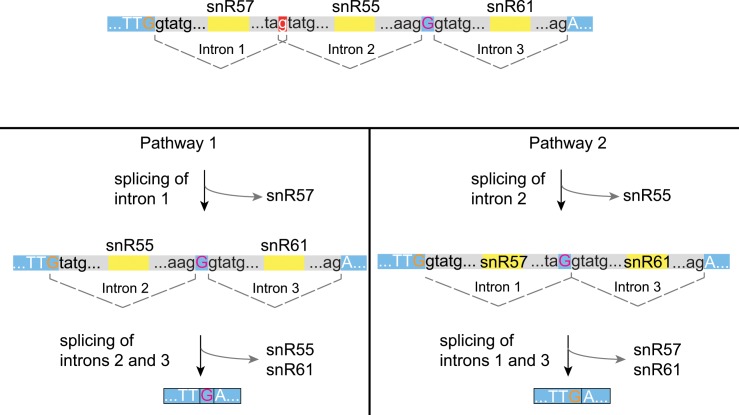
Alternative splicing of the sn57-55-61 snoRNA polycistron. Blue bars and upper case letters represent non-protein-coding exons. Introns are shown as grey bars with lower case letters. snoRNAs are shown as yellow bars. The “g” residue highlighted as a white letter on a red background can be either part of the 3’ splice junction for intron 1, or the 5’ splice junction of intron 2. The mRNA molecule is processed by one of two methods. In pathway 1, intron 1 is removed, releasing snR57 and regenerating the 5’ splice site of intron 2 by donating a G residue (highlighted in orange) from the upstream exon. Introns 2 and 3 are then spliced, releasing snR55 and snR61. The spliced product includes a one base pair exon (pink G). In pathway 2, intron 2 is removed first releasing snR55 and destroying the original 3’ splice site for intron 1. This is regenerated by donating the G residue (highlighted in pink) from the one base exon between introns 2 and 3.

In cluster II, classical, nested (internal exon of negative size) and recursive splicing (no internal exon) were observed (Fig 5). In *C*. *albicans*, splicing of this polycistron must occur in a specific order to generate snoRNAs [39]. First, the intron containing snR61 is spliced, followed by the intron containing snR57. This generates a new 5’ spice site, allowing splicing of the intron containing snR55. However, in *C*. *parapsilosis*, *C*. *orthopsilosis* and *L*. *elongisporus* the snR57-55-61 transcript can be spliced in two different ways, leading to the same outcome (Fig 5). The first pathway involves the splicing of intron 1, containing snR57. This destroys the 5’ splice site for intron 2 (snR55), while simultaneously re-generating it by donating a G residue (shown in orange) from the upstream exon (nested splicing). Introns 2 and 3, separated by a single base exon, are then spliced. In the second pathway, intron 2 is spliced first, which destroys the 3’ splice site for intron 1. This is simultaneously re-generated using the “G” from the single base exon between introns 2 and 3 (shown in pink). Introns 1 and 3 are then processed by recursive splicing (exon size of zero). The RNA-seq data indicates pathway 1 is the predominant pathway used. Luo et al [45] found that the structure of the snR57-snR55-snR61 cluster in *Debaryomyces hansenii* is the same as the species shown in Fig 5, but spicing proceeds only via pathway 1.

As described in Luo et al [45], a recombination between the snR61 and U45 snoRNAs occurred in the ancestor of the *Candida* clade, resulting in a hybrid snR61/U45 in species including *C*. *albicans*, *C*. *parapsilosis* and *L*. *elongisporus*. We confirmed that this hybrid species is also present in *C*. *orthopsilosis*.

Splicing of introns 1 and 2 in cluster III occurs by the classic mechanism in *C*. *parapsilosis*, *C*. *orthopsilosis* and *L*. *elongisporus*. The introns are separated by a single base exon. In *D*. *hansenii* processing of cluster III requires recursive splicing, as the exon size between snR41 and snR70 is zero [45].

All three snoRNAs in cluster II and five snoRNAs in cluster I are contained in introns in *Candida* species, all are intronic in filamentous fungi, and only one is exonic in *Y*. *lipolytica* [45]. In the *Saccharomyces* species however, all snoRNAs in both clusters are found in exons [45]. Mitrovich et al [39] have shown that there is a progressive reduction in intron frequency in the *Saccharomycotina*, with the intermediate loss in the *Candida* clade, and severe loss in *Saccharomyces* species.

### Processing of snoRNAs

The majority of snoRNAs within introns belong to the C/D box class, whereas most H/ACA box snoRNAs are found in exons. This organization has been described in several fungi, including *N*. *crassa* [43]. It is therefore likely that the processing mechanisms for C/D box and H/ACA box snoRNAs differ.

In *S*. *cerevisiae*, the snR78-72 snoRNAs (cluster I, Fig 3) are encoded by several exons in a single transcript. In this species, regions between the snoRNAs form stem-loop structures that are cleaved by Rnt1p [49]. The mature snoRNAs are then further processed via the exonucleolytic activity of Rat1p and Xrn1p. It is likely that many monocistronic ncRNAs in *Candida* species, such as H/ACA box snoRNAs, are processed in a similar manner. In yeast and higher eukaryotes, intronic snoRNAs are processed by exonucleases from linearized debranched lariats [50, 51]. Processing can also be independent of splicing [52]. As there are more snoRNAs within introns in *Candida* species than *S*. *cerevisiae*, splicing-related processes may be more important for maturation of C/D box snoRNAs. However, the mechanisms used for snoRNA processing in *Candida* require further analysis.

## Conclusion

We describe the identification and analysis of the non-coding landscape of three CTG-clade *Candida* species, *C*. *parapsilosis*, *C*. *orthopsilosis* and *L*. *elongisporus*, and update the *C*. *albicans* ncRNAs. We identified approximately 80 ncRNAs in each of the three species using a combination of methods, of which Infernal together with experimental validation was the most efficient. ncRNAs are highly conserved in *Candida* species. Loss and gain of snoRNAs is rare, although relocation does occur. The signal recognition particle, SCR1, RNA component of telomerase (TER1), RNase P and RNase MRP are evolving rapidly. A deletion of stem loop IV in the spliceosomal U2 RNA and loss of the associated proteins U2A and U2B occurred after the split of the *C*. *parapsilosis* species group from *L*. *elongisporus*.

## Methods

### Strains, media and RNA-seq

The strains used in this analysis were *C*. *parapsilosis* CLIB214, *C*. *orthopsilosis* CO 90–125 and *L*. *elongisporus* NRLL YB-4239. For each species, two biological replicates were grown in YPD as described in Synnott et al. [53]. RNA was extracted using the yeast RiboPure™ RNA Purification Kit. Two biological replicates were used for each strain in each condition. PolyA-selected library preparation and RNA-seq was carried out by BGI (www.genomics.cn/en) using Illumina HiSeq2500. Raw RNA-seq reads were trimmed using Skewer v0.1.117 and aligned to the respective CGOB genome using TopHat2 v2.0.12 [54, 55]. Aligned RNA-seq data was visualized using JBrowse v1.11.2 [56]. Raw RNA-seq data is available at the NCBI Sequence Read Archive using the accession number SRP077251.

### Identification of ncRNAs

Previously annotated ncRNAs from related species of yeast were compared to the genomes of *C*. *parapsilosis*, *C*. *orthopsilosis* and *L*. *elongisporus* from CGOB using BLAST [57]. The *C*. *albicans* and *S*. *cerevisiae* ncRNA tracks were downloaded from the Candida Genome Database (CGD) and Saccharomyces Genome Database (SGD) respectively [26, 27]. Ribosomal and transfer RNAs were omitted from this analysis, leaving 211 ncRNA features. BLAST results under 20 nucleotides in length or with an E-value >1E-05 were discarded. The results were manually inspected to remove fragments and duplicates.

snoRNAs were identified using Snoscan 0.9 with default *S*. *cerevisiae* methylation sites and ribosomal DNA settings, and snoGPS 0.2 with default *S*. *cerevisiae* target sites and two-stem descriptor file [29, 30]. Infernal 1.1 was used to identify RFAM family orthologs in the genomes of *C*. *parapsilosis*, *C*. *orthopsilosis*, *L*. *elongisporus* and *C*. *albicans*, accepting only the hits above the default cmscan threshold [31]. All tRNAs, rRNAs, miRNAs and poor predictions were removed manually. ncRNAs predicted by two or more different approaches or supported by RNA-seq data were retained. RNA secondary structure predictions were carried out using SFold web server [58].

## Supporting Information

S1 Fig Structure of the signal recognition particle RNA component (SCR1). (PDF)

S1 File Alignment of RNase MRP. (DOCX)

S1 Table ncRNAs in *C*. *parapsilosis*, *C*. *orthopsilois*, *L*. *elongisporus* and *C*. *albicans*. (XLSX)
