# Supplementary material for: Identification of Non-Coding RNAs in the Candida parapsilosis Species Group
Source: PLoS One. 2016 Sep 22;11(9):e0163235. doi: 10.1371/journal.pone.0163235 (PMC5033589; doi:10.1371/journal.pone.0163235)
Supplement: S1 Fig — (PDF) [file pone.0163235.s001.pdf]

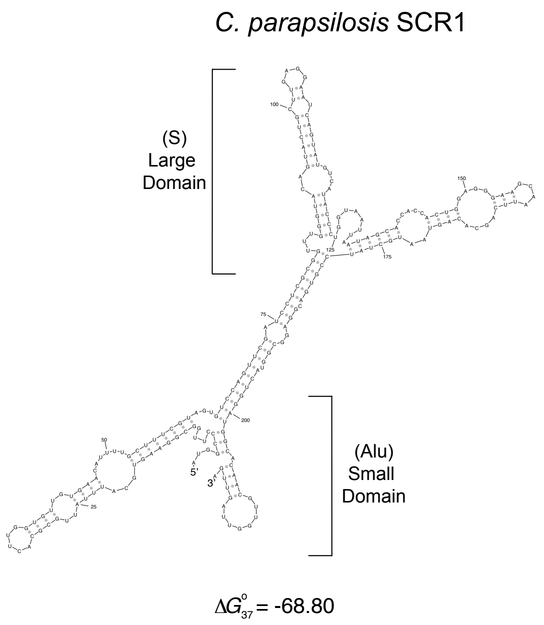


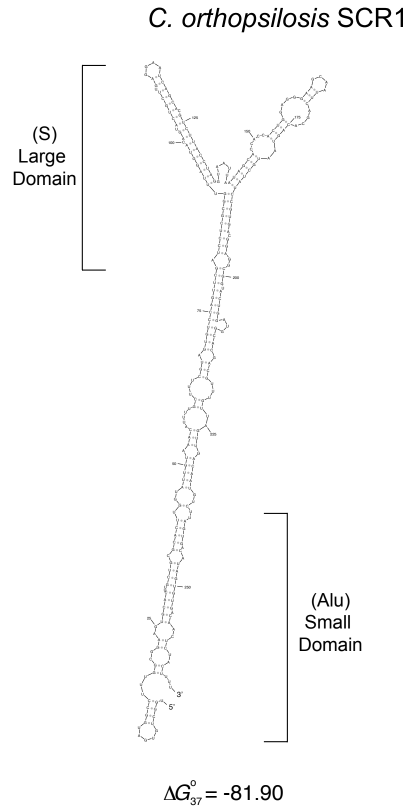


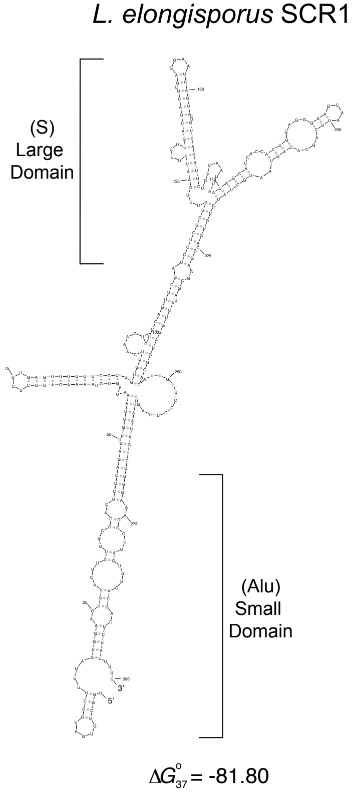


Figure S1. Structure of the signal recognition particle RNA component (SCR1)
