# Supplementary material for: Identification of Non-Coding RNAs in the Candida parapsilosis Species Group
Source: PLoS One. 2016 Sep 22;11(9):e0163235. doi: 10.1371/journal.pone.0163235 (PMC5033589; doi:10.1371/journal.pone.0163235)
Supplement: S1 File — (DOCX) [file pone.0163235.s002.docx]

File S3. Alignment of RNase MRP

Lelo = Lodderomyces elongisporus; Cpar = c. parapsilosis; Cort = C. orthopsilosis

CLUSTAL O(1.2.1) multiple sequence alignment

Lelo ---TCCATAATCAAGGAATTATCGTCGAAGCCTACAAAAGGGCATCTTTGCAGATGTCA-

Cpar ---TCCATAAACAAAGAATTGTCATCGAAGCTTACAAAAGGACATGTTTTTTCATGTCAT

Cort ACGTCCATAAACTAAGAATTGTCATCGAAGCTTACAAAAGGACATGTTTCATGT---CAT

******* * * ***** ** ******* ********* *** *** **

Lelo --------TTGTTTATAATTATAGCTTTGGGGAAAGTCTCCTGTCACAGTAGCGGAAACG

Cpar ACGCTTAATTCACTAGCGTATTTGCTTCGGGGAAAGTCTCCTGTCACAGCAGCGGAAACG

Cort ACG---CTTTAGTTAGTATTTTTGCTTTGGGGAAAGTCTCCTGTCACAACAGCGGAAACG

** ** * * **** ******************** **********

Lelo CAGCGATCTATCACTTATTTCGTTTTTAAACAATGTTTGAGCAAAACTCTGTTTGA-GAA

Cpar CGATAGAATATCATCAATTCTATTAGATGTCTTCCTTAGATTTCCAACATTGGGCCCGAA

Cort TGATAGAGTATCATCAATTCTATCGAGCATCATCCTTAAGAGCTAGCGAAATGCCCAAAT

***** *** * * ** *

Lelo TCTG--------------GAGGGACAAGGATCTCGAAAAGCTTACACTTTTTTGTGGAT-

Cpar TTCAGGTCTTTATCAGTGCTGGAGAATTGTGACTCACA----------------------

Cort TCAGGTCCGATCAGTGTTTTGGGAAAAT-TTCCCATGGAGAATCCTCTCCTACTAGGATT

* ** *

Lelo CTTGAAATCGGACAATGAAGGAAACGGTTCAGCATAGGGTC-TTCCCTATCGA-------

Cpar ---AGTATCCCCTGATTAGACGAACCTCTCGGAGGG-CATGAATGCACACCAATCCCACC

Cort GGTAGTATCCCCTGATTAGACGAACCTCGTGTGAGGGCGTGAATGCATTTGAA-------

*** ** * *** * * * *

Lelo -------TATCGAG------------TT----TGGATAGAGTTTGGTTGATTGG----TT

Cpar ATGACAGTATGGCCGTGACAAGGCTGCGCGACAAGGTTGCGCGCG-ATGTGCGGGGGGTA

Cort -------------------TC-----TCCAGTGATATTACGTATGATTGACTGGCAGGCA

* * * ** **

Lelo ATTGTTCTTCAATCCTTGAATTTTCTTTTTTGTTTTGTTGCAGCATCTTTTGTCAATTCA

Cpar TGACCCCTGCCATC-CACAACAATGGTTGA-TTTCTACTGGAAATTTTTTTTACTGGATA

Cort AGACCCCAGCAATTCAAATCCAAAGGATTA-GTTCTAATCAAAGTTTATCTGAGAATAAA

* * ** * ** * * * * * * *

Lelo TGA----------------TACCCATTTTTTTC-TTACTACTCAATTTTG-AGTCGACAA

Cpar CGT--GTGGGTGGAGGTATAGAGCAATTCGGTGGGTGCTCCAAGAATGGTTTGCTTTCAG

Cort TCTTGGTGTAATGGTGTGTTGGGCATTCCTGTAAGTCATGCCAATGTGAGTTCCTTTCAG

** * * * * * * **

Lelo TTTG----CAAAAAAAAAACACCTTTGTGTCTTTTTTTTTGTGAATTTGTTGGGCTTCTG

Cpar TTCGGTTTAAGTATCGGTCGGTCATTGGTTGATCCTTGTTGTGAGT----CGAGGCTCTG

Cort TTCG--GTGGGGCTCGGACGGTCATTGGTTGATCCTTGTTGTGAGT----CGAGATCCAG

** * * *** * * ** ****** * * * * *

Lelo TTTCAATTCTCAATTGAATCAGAAAAGCTCTGGTATTGGTGGTTTCAAT-----GGTTGA

Cpar TCGATGTTT----ATGGTTGTGTTACTAGCTCGTACGGGCCGTGTGCATCCTCGGGTGCA

Cort TTTGTCTGAGGTACTAGATATGTTACTAGCTCGTATGGAGCCGTGTCCT-----------

* * * * * * ** *** * *

Lelo TGCTTGTACTGCAAAATCAAAGCAATGGTTATTTTTGGAGTGGGACATACCGATTTGACT

Cpar CGATTGTCTGTCCCCCCGGTTGGATCGGGTGGTCAAGCTCTGGTACATGCCTAAAGCTAT

Cort TAGGACATGACTAACCCCCCTTTAATGGGTGGTTAAGCTCTGGTACTTGTCTATTTCCAC

* ** * * * *** ** * * *

Lelo TTCTGTC--CTAT--TTAACTCGTATATTGCTTGTGCCAACATTTCTTTTCAGCTGTTTG

Cpar CTGTCAGACCATGTTCTTACTGTCAAGGAGTTAGCCACACTGAATCGATTCTGCCGGTTT

Cort GGGCCAGGTCTTGGTTTTTCTTCTGAGGAGTTAGCCTCACTGAATCGATTCTGCCGTCTC

* * ** * * * ** ** *** ** * *

Lelo TG--TTTCTTGAT---------TATGAGCTGAGAGGTATAATCTTTGCCTTCCAATTTTC

Cpar CAACTGTTTGCGACCTGTGGTGTCGCCTGCGCTAGGTCTTGTCTCAATCTCTGAATCCCC

Cort AAAGCTAATTGGAGCCGCGGTGTGTCTGA---CTGGTCTTGTCTCTTCCTCCAAAAGTCC

* * *** * *** ** ** *

Lelo CTTGGACTC-----------GAGTTTGTGGAATTAGATTCCATGTGGAAGGCATTTAGTT

Cpar GCAGTTAATTGCTTACGGAGCAAAGAAAAGGATTAGCGTTTAAGTGCAGTGAACATTGTT

Cort CAAGTTACTCGTTTG-----AAACGAATGGATGGATGTATTTCGTGCAGTGAACATGCTT

* * * * *** * * * * **

Lelo T------TTAACGAAGTGCAGTCTAC--TGCTCAATGTGTTTATCAATTCCCTGTCT---

Cpar TGAATAGATACCGGTGTCCGTTG--GGTCTTGGATGATGTTTAGAGGTTCCCAGTTTGGG

Cort TGAATAGCCATCGGTGCTCGTTTTAGCCTTTGGAAGATGTTTAGAGGTTCCCAGTTAGGT

* * ** * * * * ****** ***** **

Lelo ---------------ATTGGTATTCAATTCCATACAGGATGTACACTGTCGACATTGGGG

Cpar TATTTAATTTCCCAGCTGGATGTTGTGCCCGTTTCGGGTATTACCATTTTGACAATGGGA

Cort AAG--TAATTCCCAGCTGGATGTTTTGCCCATTT-CTGGGCGGCCCTTTTGACAATGGGA

* * * ** * * * * * * **** ****

Lelo CTTTGTGTCCTGTACGAAAGTCATGATAATTGATTGAGAAGTTGTACAACTTTTCAATTA

Cpar CTTTGTGTCCTGGACCATAGTCATGACGATTGATCAATT--------------TCGATCA

Cort CTTTGTGTCCTGGACGAAAGTTATGACGATTGATCAATT--------------ACGATCA

************ ** * *** **** ****** * * ** *

Lelo ACAGAATGGAGCTTAGCCTTGTATTATGG---

Cpar ACCCAATGGAGCTTACTCTTGTTTTATGG---

Cort ACCCAATGGAGCTTACTCTTACTTTATGGACG

** *********** *** ******
